# Serum from dengue virus-infected patients with and without plasma leakage differentially affects endothelial cells barrier function *in vitro*

**DOI:** 10.1371/journal.pone.0178820

**Published:** 2017-06-06

**Authors:** Francielle Tramontini Gomes de Sousa Cardozo, Gyulnar Baimukanova, Marion Christine Lanteri, Sheila Marie Keating, Frederico Moraes Ferreira, John Heitman, Cláudio Sérgio Pannuti, Shibani Pati, Camila Malta Romano, Ester Cerdeira Sabino

**Affiliations:** 1 Department of Infectious and Parasitic Diseases, Institute of Tropical Medicine, University of São Paulo, São Paulo, São Paulo, Brazil; 2 Blood Systems Research Institute, BSRI, San Francisco, California, United States; 3 Department of Laboratory Medicine, University of California, San Francisco, California, United States; 4 University of São Paulo School of Medicine, Division of Immunology - Heart Institute, São Paulo, São Paulo, Brazil; 5 University of Santo Amaro, São Paulo, São Paulo, Brazil; University of Hong Kong, HONG KONG

## Abstract

**Background:**

Although most of cases of dengue infections are asymptomatic or mild symptomatic some individuals present warning signs progressing to severe dengue in which plasma leakage is a hallmark.

**Methodology/Principal findings:**

The present study used Electric Cell-substrate Impedance Sensing (ECIS^®^) which allows for electrical monitoring of cellular barrier function measuring changes in Transendothelial Electric Resistance (TEER) to investigate the parameters associated with dengue induced leakage. Three groups of individuals were tested: dengue-positives with plasma leakage (leakage), dengue-positives without plasma leakage (no leakage), and dengue-negatives (control). Data show that TEER values of human umbilical vein endothelial cells (HUVECs) was significantly lower after incubation with serum from subjects of the leakage group in comparison to the no leakage or control groups. The serum levels of CXCL1, EGF, eotaxin, IFN-γ, sCD40L, and platelets were significantly decreased in the leakage group, while IL-10, IL-6, and IP-10 levels were significantly increased. We also found a strong correlation between TEER values and augmented levels of IP-10, GM-CSF, IL-1α, and IL-8, as well as decreased levels of CXCL1 and platelets.

**Conclusions/Significance:**

The present work shows that the magnitude of the immune response contributes to the adverse plasma leakage outcomes in patients and that serum components are important mediators of changes in endothelial homeostasis during dengue infections. In particular, the increased levels of IP-10 and the decreased levels of CXCL1 and platelets seem to play a significant role in the disruption of vascular endothelium associated with leakage outcomes after DENV infection. These findings may have important implications for both diagnostic and therapeutic approaches to predict and mitigate vascular permeabilization in those experiencing the most severe clinical disease outcomes after dengue infection.

## Introduction

Brazil is a dengue-endemic country where peaks of dengue virus (DENV) transmission have occurred in 2002, 2008, 2010, and 2013. In 2015 and in 2016 Brazil has reported about 1.5 probable cases with respectively 21.591 and 8.237 confirmed cases of dengue with warning signs [1]. Estimates indicate that about 1% of people infected with DENV worldwide are diagnosed with severe dengue. Of those, fatality rates are approximately 4% [2].

An adverse outcome of the inflammation triggered in response to DENV infection is the exudation of plasma proteins and fluid into the interstitial compartment resulting in depleted intravascular volume and hypotension, which may evolve into hypovolemic shock. Along with coagulopathy, plasma leakage is a significant pathological hallmark of severe dengue and is frequently associated with dysfunction of endothelial microvasculature especially in the lungs and the abdominal cavity where it results in pleural effusion and ascites [3, 4].

Electric Cell-substrate Impedance Sensing (ECIS^®^) is a tool to electrically monitor cell behavior in tissue culture by measuring changes in Transendothelial Electric Resistance (TEER), a proxy for endothelial barrier function [5]. A reduction on TEER corresponds to a decrease in cellular integrity and an increase in paracellular permeability [6].

In this work we sought to assess the effect of serum collected from leakage versus non leakage DENV infected patients on the in vitro endothelial barrier function through the ECIS^®^ system. The obtained results showed that the contact of HUVECs with serum from DENV-positive patients with clinical signs of leakage resulted in significant lower TEER values in comparison to that from DENV-positive patients without leakage. This finding supports the hypothesis that serum components are likely to contribute to changes in endothelial cell behavior. The concentrations of 38 immunomediators were then measured in the serum samples in an attempt to identify potential mediators of endothelial barrier disruption.

## Methods

### Study participants

Samples from DENV-infected patients were collected in Santos, São Paulo state, Brazil during the 2010 dengue outbreak which occurred from February through May, 2010. Individuals who presented with dengue-like symptoms at the Ana Costa Hospital of Santos were evaluated by a doctor, answered a symptom questionnaire, and had signed a consent form term. Blood samples were collected from acutely ill patients at the time of hospital admission and before any treatment intervention or diagnostic procedure. Samples collected between 2 and 7 days after symptoms onset were selected for the present study, which was approved by the Institutional Review Board from Hospital das Clínicas, University of São Paulo (CAPPesq), approval number 0652/09.

Laboratory confirmation of DENV infection was performed through the detection of NS1 antigen and IgM/IgG-specific antibodies using a rapid test (Dengue duo test bioeasy, Standard Diagnostic Inc.) and detection of DENV RNA by RT-qPCR, as described elsewhere [7]. DENV-positive patients were defined as having laboratory-confirmed dengue if the RT-qPCR and/or NS1 assays were positive at enrollment. The classification of primary or secondary infections was determined by an antibody avidity test [8]. Plasma leakage was identified by physical examination and confirmed by ultrasound or X-ray. Sera from healthy blood donors which were DENV RNA negative by the transcription-mediated amplification (TMA) test (Hologic Inc.) were used as control.

### Evaluation of endothelial barrier function in vitro

To evaluate the putative effects of serum collected from DENV infected patients on TEER, HUVECs (Human Umbilical Vein Endothelial Cells, Lonza, Walkersville, USA) were seeded (5x10^4^ cells/well) in L-cysteine reduced ECIS 96W1E+ arrays and monitored in the ECIS system 1600 (Applied BioPhysics, Troy, USA). After 24 h of incubation at 37°C and 5% CO_2_, cells were starved in EBM^™^-2 basal medium without supplementation and then incubated in medium with 10% human serum collected from DENV-positive patients with leakage, DENV-positive patients without leakage, or DENV-negative blood donors. Due to limited sample volume available, only 13 samples per group were tested in this assay. For baseline control condition, cells received EBM^™^-2 complete medium only. EBM^™^-2 basal medium supplemented with vascular endothelial growth factor (VEGF) at 50 ng/mL was used as a positive control [9]. Data, recorded at 4 kHz, were collected real-time throughout 24 h and analyzed using ECIS Software v1.2.92.0 [10].

### Multiplex analysis for serum proteins

The concentrations of 38 analytes were measured in serum samples using the MILLIPLEX^®^ MAP Human Cytokine/Chemokine Magnetic Bead Panel (HCYTMAG-60K-PX38; EMD Millipore, Hayward, USA) with standard curves ranging from 3.2 to 1x10^4^ pg/mL. Subjects’ serum samples and standard curves were run in duplicate following the manufacturer’s protocols. Fluorescence signals were detected using the multiplex array reader Luminex^®^ 200^™^ System (Luminex Corporation, Texas, USA) and analyzed using the Bio-Plex manager 6.1 software (Bio-Rad, California, USA). Results that were outside of the lowest range of the standard curve were set as half the lowest value in the set for the respective analyte. The values that were outside the highest range were replaced with the highest value in the set.

### Systems analysis

QIAGEN’s Ingenuity^®^ Pathway Analysis (IPA^®^, QIAGEN Redwood City, http://www.qiagen.com/ingenuity) was used to perform core analyses in order to compare results of immunomediators quantification. Data sets with the gene identifiers of the 38 analytes and the corresponding fold changes of mean levels of leakage group in relation to the no leakage group were used in the analysis.

### Albumin quantification

Serum albumin concentrations were determined by the bromocresol purple albumin assay kit (Sigma Aldrich, Missouri, USA) according to the manufacturer’s instructions.

### Statistical analysis

TEER values were obtained in two experiments, each with 6–8 serum samples per group, in quadruplicate. Resistance measurements were normalized by dividing time-point data by each well’s baseline resistance. “% Change of Normalized Resistance”, calculated at both 30 and 120 min after treatment, refers to change from measurements obtained prior to treatment, also adjusted by subtracting average changes in control wells receiving media-only. Medians were averaged to create a group mean for each of the three groups.

Statistical analyses were performed using R (version 3.0.2). Data were analyzed by normality tests and then parametric (t-test or One-way ANOVA and Tukey's multiple comparison post-test) or non-parametric (Mann Whitney or Kolmogorov-Smirnov) tests were used to compare groups. Correlation analyses were performed by two-tailed Pearson test. The raw fluorescence intensities obtained in the bead-based immunoassay were submitted to the quantile normalization. Differential expressed cytokine levels were determined by a moderated t-test implemented in the LIMMA R package [11]. The Principal Component Analysis was performed using the FactorMineR with the R package [12]. The z-score hierarchical clustering was carried out using the Manhattan distance measure and Ward's method for linkage analysis. In all tests statistical significance threshold was *p*<0.05.

## Results

### Description of patient data

Clinical and laboratorial data of individuals from the control (healthy blood donors), no leakage (DENV infected patients without signs of leakage), and leakage (DENV infected patients with clinical signs of leakage) groups were compiled in Table 1.

**Table 1:** Clinical and laboratory data of study participants.

	Control (n = 31)	No leakage (n = 29)	Leakage (n = 28)	*p* value(No leakage vs Leakage)
**Age**^a^^,^^b^	43 (22–56)	36 (18–77)	14 (1–62)	*p = 0.0322
**Age groups**^c^				
**0–5**	0	0	2	-
**6–15**	0	0	13	-
**16–25**	4	5	1	-
**26–60**	27	22	11	-
**>60**	0	2	1	-
**Gender (male)**^d^	19 (61.29%)	11 (37.93%)	13 (46.43%)	ns *p* = 0.4595
**Platelet number x 10**^**3**^**/mm**^**3**^ **of blood**^a^	nd	156 (26–271)	36 (10–167)	*p<0.0001
**Days of symptoms**^a^	-	3 (3–4)	4 (2–7)	ns *p* = 0.1558
Distribution of days of symptoms^e^				
2	-	0	1	-
3	-	11	11	-
4	-	18	8	-
5	-	0	5	-
6	-	0	2	-
7	-	0	1	-
**Viral Load (genomic copies/mL)**^a^	-	1.06 x 10^3^(81–2.22 x 10^7^)	1.82 x 10^2^(73–9.24 x 10^6^)	ns *p* = 0.0695
**IgG avidity**	-	18 (62.07%) secondary	27 (96.43%) secondary	ns *p* = 0.0692
		12 (37.93%) not detected	1 (3.57%) not detected	
**NS1+ / RNA+ /IgM+**	-	3	4	-
**NS1+ / RNA+ /IgM-**	-	10	1	-
**NS1+ / RNA- /IgM-**	-	1	0	-
**NS1- / RNA+ /IgM-**	-	9	2	-
**NS1- / RNA+ /IgM+**		3	15	-
**NS1+ / RNA- /IgM+**		3	6	-
**Plasma leakage classification**	-	-	8 (28.57%) Peritoneal	-
			4 (14.28%) Pleural	
			9 (32.14%) Pleural and Peritoneal	
			3 (10.71%) Peritoneal/Shock	
			4 (14.28%) Pleural/Shock	
**Shock**	-	0 (0%)	7 (25.00%)	*p = 0.0039
**Dead**	-	0 (0%)	2 (7.14%)	ns *p*>0.9999
**Hemorrhagic manifestations**^f^	-	0 (0%)	16 (57.14%)	*p = 0.0002

Abbreviations: nd, not determined; ns, not significant.

Values are given in number of patients (percentage of total).

^a^ Age, platelet number, days of symptoms, and viral load are given in median (minimum-maximum).

^b^ median age values were significantly different between control and leakage groups (*p* = 0.0009). No statistical differences were found between control and no leakage groups.

^c^ Distribution of age groups.

^d^ No statistical differences were found for gender between control and no leakage groups or between control and leakage groups.

^e^ Distribution of days of symptoms: All the samples were collected upon hospital entrance and the day after symptom onset was calculated based on symptomatology described by the patient during the anamnesis. Only samples which were collected at days 2–7 after first symptom were selected to the current study.

^f^ Hemorrhagic manifestations included petechiae, epistaxis, gingival bleeding, menorrhagia, hematuria, gastrointestinal, and/or pulmonary bleeding.

*Indicates statistical significance with *p*<0.05.

### Evaluation of TEER of endothelial cells

**Fig 1:**
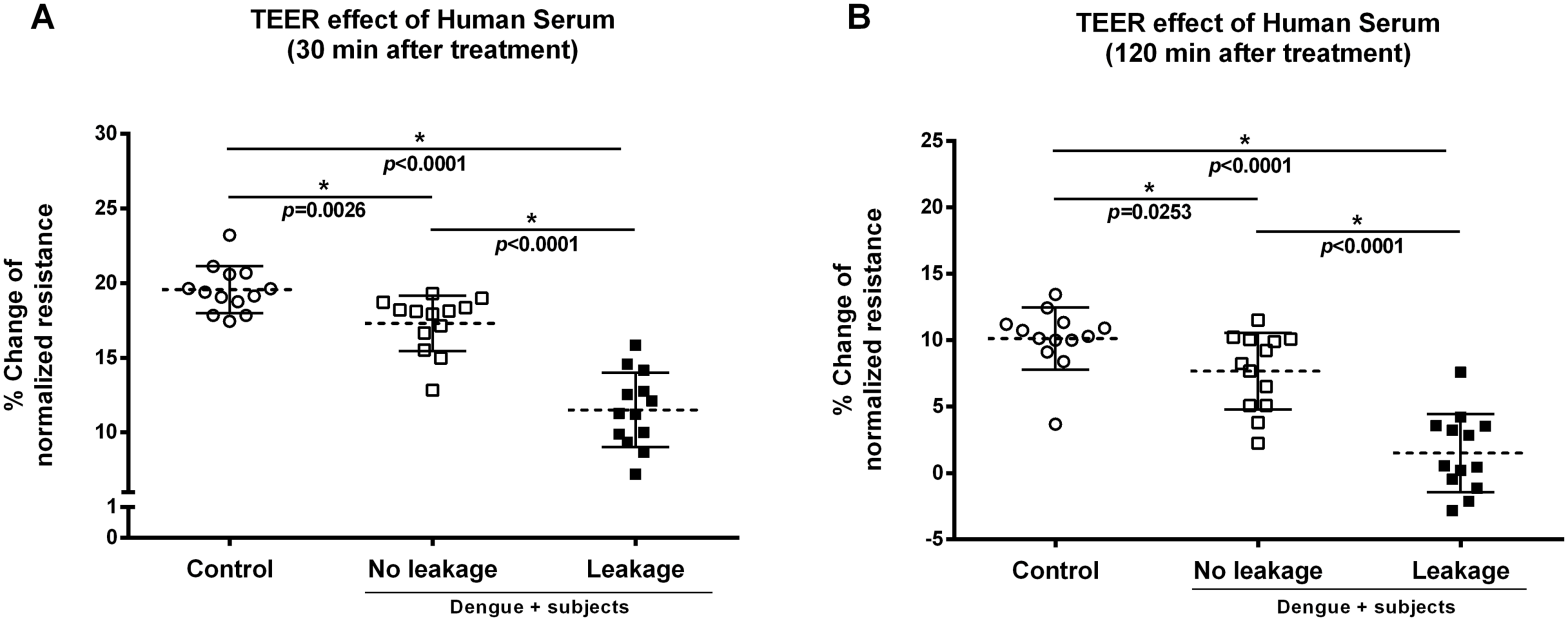
Effect of serum from DENV-positive patients on TEER of endothelial cells. Confluent monolayers of HUVECs cultured in ECIS arrays were treated or not with 10% serum from three different groups: Healthy blood donors (control, n = 13), DENV infected patients without leakage (no leakage, n = 13), or DENV infected patients with leakage (leakage, n = 13). (*) Asterisks indicate statistically significant differences (ANOVA/ Tukey’s test) between groups with *p*<0.05. TEER values were obtained at 30 (A) and 120 (B) min after treatment.

Fig 1 shows that cells treated with samples from both DENV-positive groups displayed TEER values significantly lower than the normal control group. The mean increase in resistance for the leakage group was significantly smaller than the mean resistance increases for either of the other two groups (control and no leakage), at 30 and 120 min post-treatment period time points.

**Fig 2:**
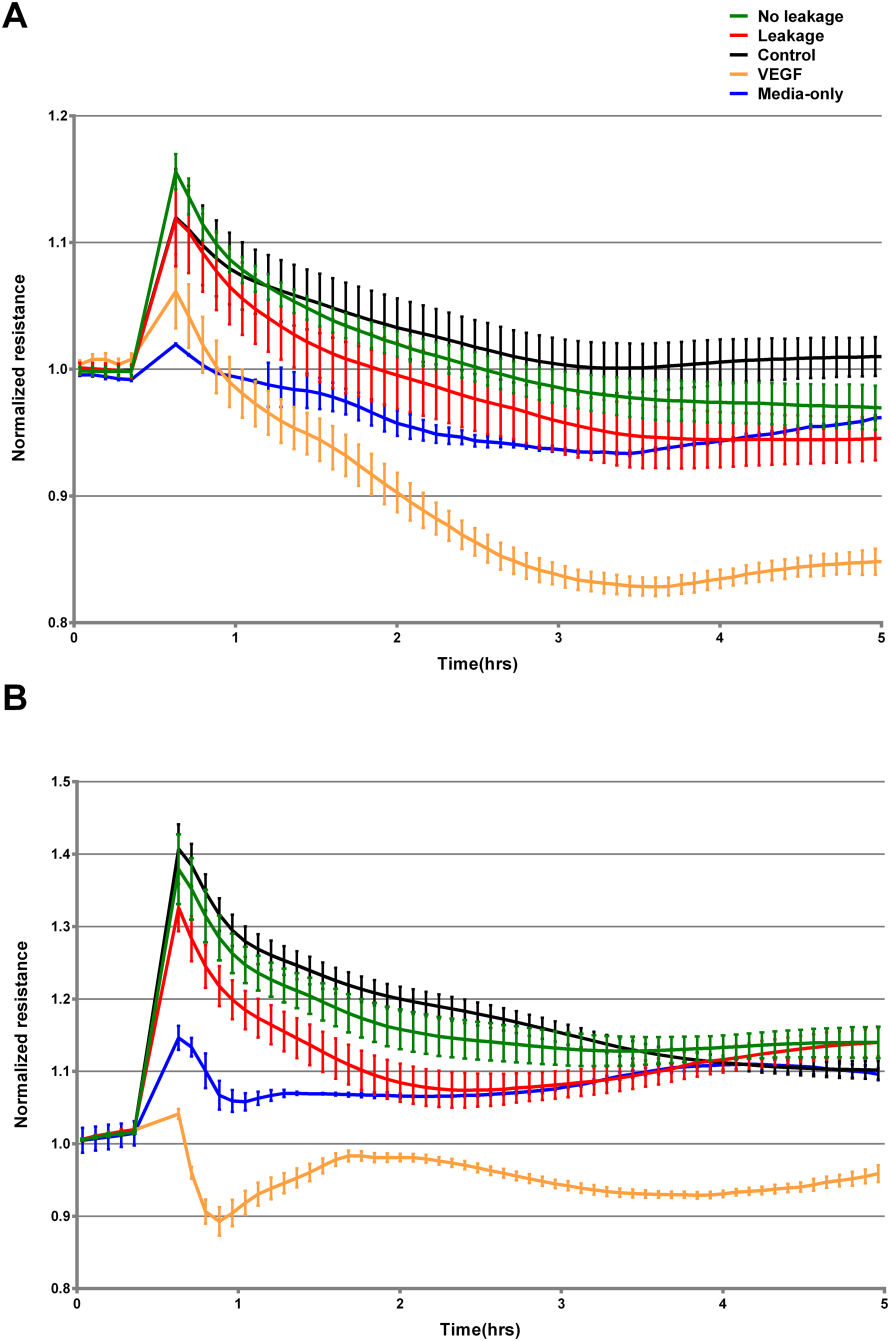
Real-time impedance measurement throughout 5 h after treatment. Confluent monolayers of HUVECs cultured in ECIS arrays were treated or not with 10% serum from three different groups: Healthy blood donors (control, n = 13), DENV infected patients without leakage (no leakage, n = 13), or DENV infected patients with leakage (leakage, n = 13). Graph A shows results from the first experiment and graph B from the second experiment.

Fig 2 shows normalized TEER results in traces collected by ECIS through 5 h, starting 30 min before addition of serum samples until 4.5 h after treatment.

The Kolmogorov-Smirnov test showed no significant differences in viral load between the DENV infected groups (Table 1). Moreover, no correlation between viral load and TEER 30 min (*r* = -0.24) or TEER 120 min (*r* = -0.31) values was found.

**Fig 3:**
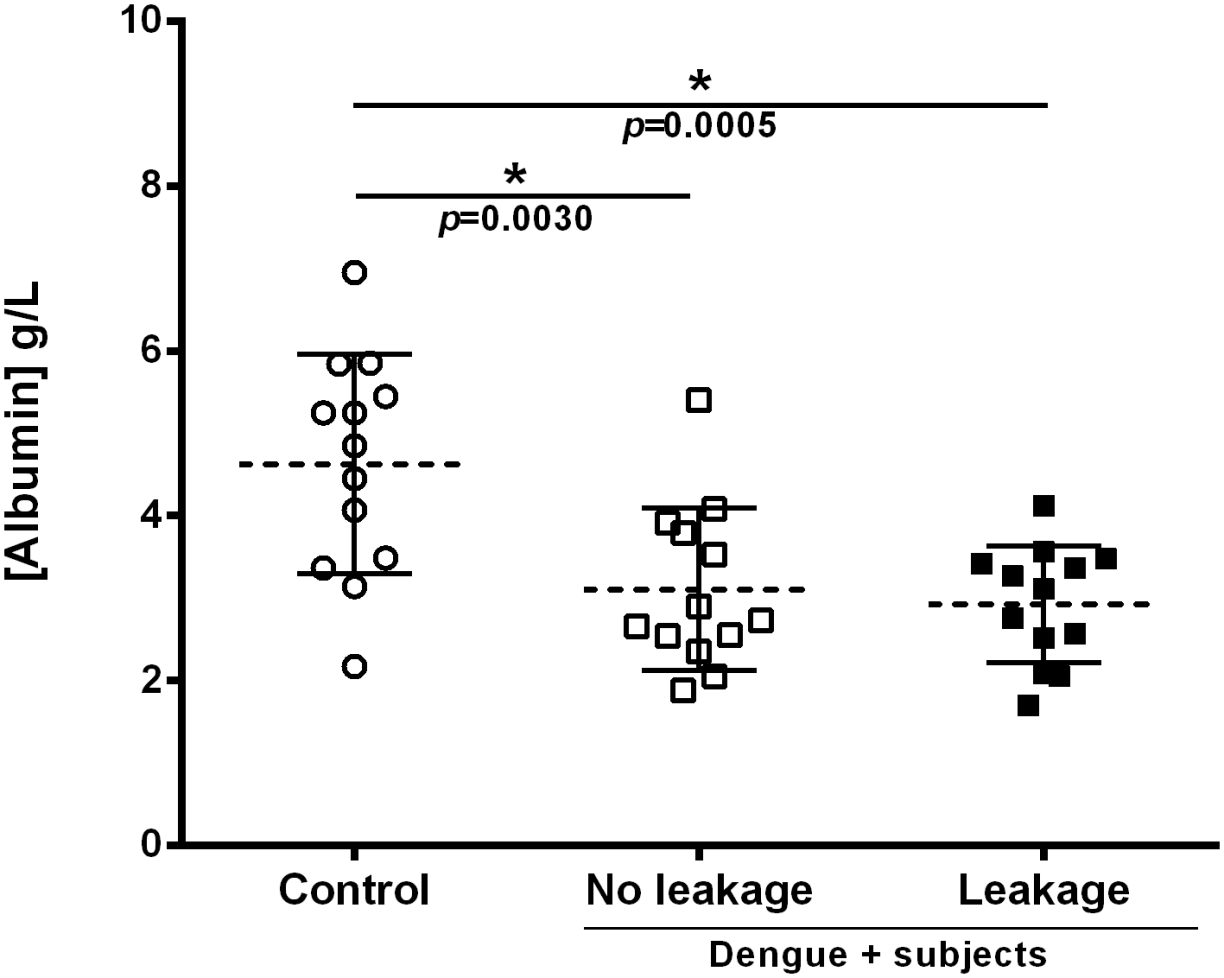
Serum albumin levels. Albumin levels of serum from the three different groups: Healthy blood donors (control, n = 13), DENV infected patients without leakage (no leakage, n = 13), or DENV infected patients with leakage (leakage, n = 13) were quantified by the bromocresol purple method. Groups were compared by the ANOVA/Tukey’s test. *Statistically significant difference (*p*<0.05).

The ANOVA/Tukey’s test, used to compare albumin levels among groups, showed statistical differences between infected groups and control (Fig 3).

We also then normalized the % of normalized resistance values by dividing them by the corresponding albumin level of each sample. As a result, the leakage group still presented significant lower resistance values in comparison to the no leakage group at 30 min time-point. For the 120 min time-point, the leakage group had significant lower resistance values in comparison to the no leakage and control groups (S1 Fig). Moreover, since we found no statistical difference in albumin levels between the no leakage and leakage groups and no correlation between albumin levels and TEER 30 min (*r* = 0.45) or TEER 120 min (*r* = 0.32) values, we conclude that the differences in TEER readings do not seem to be an effect of serum albumin concentration.

A positive correlation between platelets numbers and TEER changes at 30 (*r* = 0.63, *p* = 0.001) or 120 min (*r* = 0.53, *p* = 0.006) was observed.

### Quantification of immune components in serum from control, no leakage, and leakage distinct groups

**Fig 4:**
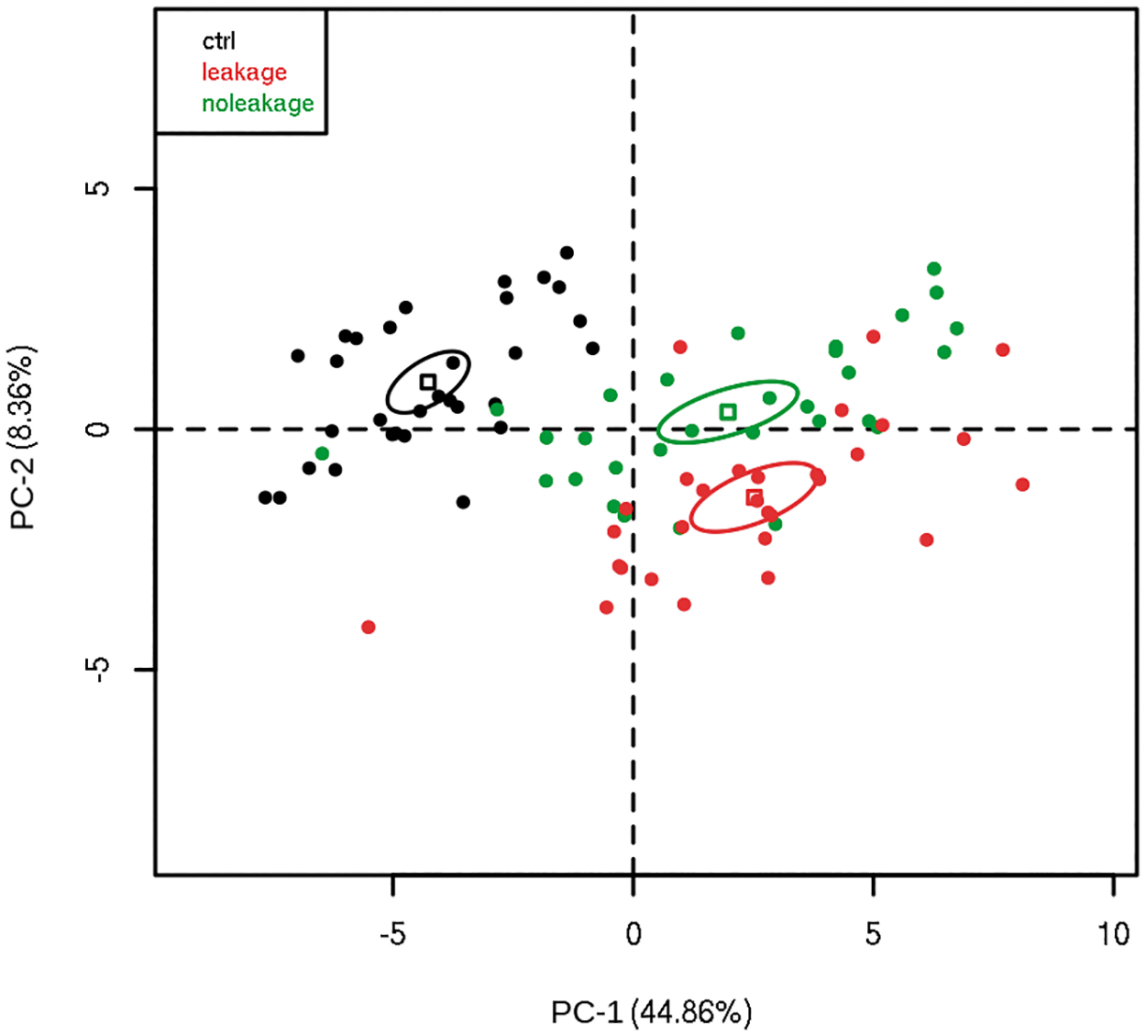
Principal component analysis plot of immunomediators levels. Serum samples from the following groups: healthy blood donors (control, n = 31), DENV infected patients without signs of plasma leakage (no leakage, n = 29), and DENV infected patients with signs of plasma leakage (leakage, n = 28) were assessed for 38 analytes, including cytokines, chemokines, and growth factors. The small square is the geometric center of each distribution and the ellipse represents the groups’ dispersion.

Principal Component Analysis (PCA) of the expression data revealed a good segregation of the samples according to the three studied groups (Fig 4). PCA is a vectorial space reduction or an orthogonal transformation performed in a set of possibly correlated observed variables into linear independent set of variables of highest variance, the principal components. Samples belonging to the same groups usually share a similar variance and tend to cluster together when projected on the plane of principal components.

Quantification of 38 analytes related to immune response in serum samples from each group revealed significant augmented levels of IL-12p40, IL-1α, and MCP-1, as well as reduced levels of FGF-2, IL-3, IL-7, and MIP-1β in serum from DENV-positive patients in comparison to healthy controls, with no difference between the leakage and no leakage groups. In contrast, levels of Fractalkine, GCSF, GM-CSF, IFN-α, IL-12p70, IL-13, IL-1β, IL-1RA, IL-2, IL-8, IL-9, MDC, MIP-1α, and TGF-α were similar among the three groups. Interestingly, the levels of CXCL1, EGF, eotaxin, IFN-γ, IL-10, IL-6, IP-10, and sCD40L were significantly different when comparing the leakage and no leakage groups (Table 2).

**Table 2:** Comparison of immune components levels in serum from control, no leakage, and leakage groups.

Analyte	No Leakage vs Control		Leakage vs Control		Leakage vs No Leakage	
	Fold change	adjusted *p* value	Fold change	adjusted *p* value	Fold change	adjusted *p* value
CXCL1	-5.02	<0.001	-11.04	<0.001	-2.20	<0.001
EGF	-3.67	<0.001	-13.39	<0.001	-3.64	<0.001
Eotaxin (CCL11)	-1.13	0.463	-1.81	<0.001	-1.60	0.013
FGF-2	-1.77	<0.001	-1.63	<0.001	1.08	0.616
FLT-3L	-2.09	0.035	-1.87	0.068	1.12	0.914
Fractalkine (CX3CL1)	1.07	0.614	1.16	0.250	1.09	0.657
GCSF (CSF3)	1.03	0.793	1.14	0.261	1.11	0.488
GM-CSF (CSF2)	-1.18	0.187	-1.15	0.253	1.02	0.918
IFN-α2	-1.07	0.614	-1.04	0.799	1.04	0.918
IFN-γ	1.50	0.017	-1.15	0.433	-1.74	0.003
IL-10	15.36	<0.001	51.51	<0.001	3.35	0.020
IL-12p40 (IL-12β)	17.03	<0.001	32.14	<0.001	1.89	0.488
IL-12p70	-1.37	0.292	-1.59	0.097	-1.16	0.765
IL-13	-1.11	0.786	1.01	0.977	1.12	0.916
IL-15	1.43	0.405	2.35	0.030	1.65	0.366
IL-17	1.46	0.405	-1.14	0.791	-1.67	0.366
IL-1α	8.26	<0.001	13.38	<0.001	1.62	0.059
IL-1β	-1.39	0.405	-1.12	0.791	1.24	0.743
IL-1RA	-1.37	0.145	-1.07	0.791	1.28	0.366
IL-2	1.13	0.708	1.22	0.613	1.07	0.918
IL-3	-7.58	<0.001	-7.63	<0.001	-1.01	0.991
IL-4	-2.27	0.072	-5.64	<0.001	-2.49	0.075
IL-5	-2.95	0.015	-2.97	0.013	-1.00	0.991
IL-6	1.36	0.463	3.69	0.001	2.71	0.032
IL-7	-2.00	0.001	-2.54	<0.001	-1.27	0.366
IL-8 (CXCL8)	-1.09	0.696	1.13	0.613	1.23	0.488
IL-9	1.85	0.118	1.64	0.216	-1.13	0.914
IP-10 (CXCL10)	8.57	0.001	266.83	<0.001	31.12	<0.001
MCP-1 (CCL2)	2.72	<0.001	2.45	<0.001	-1.11	0.766
MCP-3 (CCL7)	-2.04	0.002	-1.50	0.090	1.36	0.366
MDC (CCL22)	-1.43	0.072	-1.13	0.569	1.26	0.366
MIP-1α (CCL3)	-1.32	0.292	-1.28	0.337	1.03	0.948
MIP-1β (CCL4)	-1.47	0.001	-1.81	<0.001	-1.23	0.165
sCD40L	-1.67	0.463	-52.74	<0.001	-31.62	<0.001
TGF-α	2.08	0.074	-1.03	0.967	-2.13	0.113
TNF-α	-2.07	<0.001	-1.40	0.053	1.47	0.058
TNF-β	-1.91	0.072	-2.03	0.044	-1.06	0.918
VEGF (VEGFA)	1.14	0.292	1.27	0.041	1.12	0.488

Results of quantification of each individual serum sample were used to calculate mean of the three groups. Groups were compared each other by fold change. Results with statistical significant differences (*p*<0.05) were highlighted in grey.

S2 Fig. illustrates the expression profile of the eight immunomediators that were significantly different among the leakage and no leakage groups.

Data from TEER and serum protein levels were used to perform Pearson correlation tests. Considering the 30 min time point, TEER values had strong positive correlation with CXCL1 (*p*<0.0001, *r* = 0.79), and negative correlation with GM-CSF (*p*<0.0001, *r* = -0.67), IL-1α (*p*<0.0001, *r* = -0.69), IL-8 (*p*<0.0001, *r* = -0.58), and IP-10 (*p*<0.0001, *r* = -0.63). Regarding 120 min time point, TEER values displayed strong positive correlation with CXCL1 (*p*<0.0001, *r* = 0.68), and strong negative correlation with GM-CSF (*p*<0.0001, *r* = -0.65), IL-1α (*p*<0.0001, *r* = -0.62), IL-8 (*p*<0.0001, *r* = -0.52), and IP-10 (*p*<0.0001, *r* = -0.54) (S3 Fig).

**Fig 5:**
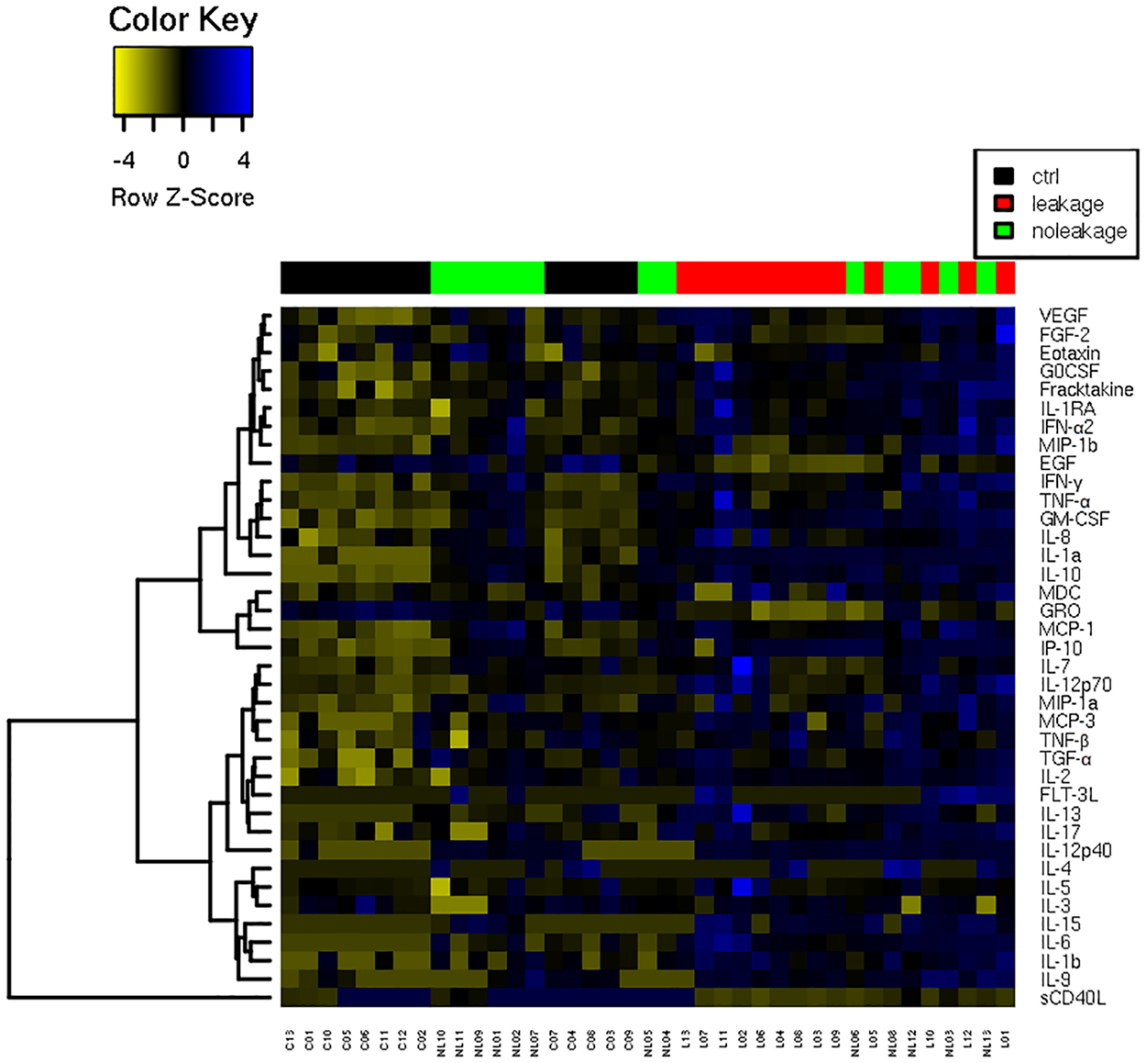
Heat map. Z-score hierarchical clustering based on squared Euclidean distance measure and Ward's method for linkage analysis. Each row represents one of the 38 cytokines and each column represents one sample. Samples from control (C, n = 13) no leakage (NL, n = 13) or leakage (L, n = 13) groups were arranged from left to right in descending order of TEER values. The color scale means the gene expression standard deviations from the mean, with yellow for low expression and blue for the high expression levels.

Cluster analyses of the cytokines expression levels showed that the samples were satisfactory segregated into groups according to their corresponding expression profile (Fig 5). The leakage samples regrouped on the right and the control samples regrouped on the left while the no-leakage samples regrouped in the center of the panel between the other two groups.

## Discussion

Several previous studies have focused on determining cytokine levels in serum of DENV infected patients, comparing groups according to the 1997 or 2009 WHO case classification [13]. Herein, we focused in the plasma leakage warning sign including DENV-positive groups with and without plasma leakage, independently of case classification, in order to better correlate clinical data with endothelial cell function in vitro. Serum samples from DENV-positive symptomatic patients were collected in the acute phase of the disease, from days 2 to 7 after symptom onset. Patient demographic data, viral load, albuminemia, and serum immune components were evaluated and linked with TEER data, as a sensitive parameter of in vitro endothelial barrier integrity. This approach brought some new aspects about factors that might be associated with dengue vascular disorders.

DENV infections are characterized by extensive immune activation with production of high levels of immunomediators mostly with proinflammatory properties. The more accepted mechanism of plasma leakage involves the interaction of viral and host circulating factors (for instance, chemokines, cytokines, growth factors, NS1, and antibodies) with endothelia causing endothelial barrier function modifications, including upregulation of ICAM-1 and VCAM-1 expression, that increases permeability [14–16]. The present study shows that TEER values in the leakage group were significantly lower than the no leakage and control groups, supporting the importance of serum components in the endothelial activation. It is important to mention that due the higher complexity of human serum in relation to the basal EBM-2 medium the addition of serum to cell monolayers causes enhancement on TEER readings. Therefore increased TEER values were measured in all the three serum treated groups, with significant lower TEER readings detected in the leakage group.

Statistical analysis indicated that eight analytes had significant differences between the leakage and no leakage groups. The levels of CXCL1, EGF, eotaxin, IFN-γ, and sCD40L were decreased in the serum from the leakage group, while IL-10, IL-6, and IP-10 levels were increased (see Table 2).

CXCL1 is a member of the CXC chemokine family expressed by platelets, endothelial cells, neutrophils, monocytes, and macrophages. CXCL1 has neutrophil chemoattractant, angiogenic, proinflammatory, and tumorigenic activities. By signaling through the chemokine receptor CXCR2 it participates in monocyte arrest and adhesion to endothelial cells [17]. Our results show that the leakage group displayed significantly lower levels of CXCL1 when compared to both no leakage and control groups while the no leakage group presented significantly lower CXCL1 levels in comparison to control group. The DENV-induced thrombocytopenia caused by impaired thrombopoiesis and peripheral destruction of platelets may explain the reduced CXCL1 serum levels since platelets represent a source of this chemokine [18]. In fact, we found a very strong positive correlation (*p*<0.0001, *r* = 0.80) between platelets counts and CXCL1 levels. As mentioned before, we also detected a strong positive correlation between CXCL1 serum concentration and TEER values. This finding led us to consider an endothelial protective effect of this chemokine. Despite the often described proinflammatory and chemoattractive activities of CXCL1, Liehn and colleagues [19] have shown that the keratinocyte-derived chemokine (KC), the mouse orthologue of human CXCL1, promoted the endothelial recovery after vascular injury via CXCR2 in mice. These authors suggested that KC function on endothelial recovery may outweigh its function on monocyte or neutrophil recruitment. It is also important to mention that CXCL1 was previously shown to have in vitro proliferative and angiogenic activities in HUVECs cultures [20, 21]. Therefore, despite lower concentrations, the CXCL1 present in serum samples could have interfered with HUVECs behavior in our experiments resulting in augmented TEER values. The potential protective effect of CXCL1 and its relationship with vascular permeability remains to be determined.

The epidermal growth factor (EGF) levels were significantly reduced in the DENV infected groups in comparison to the control. The leakage group had the lowest EGF levels in comparison to the other groups. Decreased levels of EGF, analyzed in samples collected in the same dengue outbreak, were previously associated with severe illness (shock cases) [22]. These authors suggested that depletion of this growth factor might be due the thrombocytopenia since thrombocytes are an important source of EGF. The strong positive correlation (*p*<0.0001, *r* = 0.61) we found between patient’s platelets counts and EGF levels corroborate this. Alternatively, the reduced EGF levels in severe dengue patients (Dengue Hemorrhagic Fever- DHF) might reflect its consumption in the endothelial repair process [23].

Eotaxin (CCL11) is a proinflammatory chemokine that elicits chemotaxis especially of eosinophils, and also basophils, mast cells, and Th2 lymphocytes. Eotaxin was shown to suppress the in vitro secretion of IL-8 (CXCL8) by TNF-α-stimulated endothelial cells via CCR3 [24]. We found that the no leakage group had higher levels of eotaxin in comparison to the leakage and control groups. Nevertheless, Rathakrishnan et al. [25] reported decreased eotaxin levels in DENV-positive patients without warning signs in comparison to DENV-positive patients with warning signs.

Interferon gamma (IFN-γ) is produced by CD4+ T cells, during T-lymphocyte helper type 1 (Th1) response to viral antigens during host antiviral state [26]. The relationship between elevated levels of IFN-γ and dengue severity is not clear since discrepant findings have been reported. IFN-γ levels were significantly increased in serum or plasma of patients with severe dengue when compared to mild disease forms [27–29]. In contrast, Ferreira et al. [30] did not detect statistical significant differences in IFN-γ levels of DHF group in comparison to dengue fever (DF) group in young Brazilian patients during the 2008 epidemic. In the present study, an increased amount of IFN-γ was detected in both DENV-positive groups and the no leakage group had significantly higher serum levels. IFN-γ may have a role in protecting patients from severe dengue since it triggers pathways of innate, such as NK activation and production of IFN-α and nitric oxide, and adaptive cellular immunity, by inducing antigen presentation and apoptosis, both essential to control DENV replication and overcome infection [14, 30, 31].

Soluble CD40 ligand (sCD40L) is a protein fragment resulting of the cleavage of CD40L, a trimeric transmembrane protein highly expressed in activated platelets [32]. Given its proinflammatory activity we would expect elevated levels of sCD40L in DENV-positive patients. However, the leakage group displayed significant lower levels of sCD40L in comparison to the other two groups. Likewise, McElroy and colleagues [33] unexpectedly found increased sCD40L levels in nonfatal cases of Ebola hemorrhagic fever. It is important to mention that we had 18 individuals with sCD40L levels above the assay detection limit. Analyzing the data set without these out of range results we found a positive correlation (p<0.001, r = 0.53) between sCD40L levels and platelet counts, which might indicate that lower sCD40L levels is a result of platelets depletion.

IL-10 is secreted by different cell types including CD4+ and CD8+ T cells, B cells, macrophages, monocytes, eosinophils, and mast cells. High levels of IL-10 in serum have been previously associated with disease severity on dengue infections probably by suppression of dengue-specific T cell responses [revised by Lee et al. [34]]. IL-6 is an acute-phase cytokine secreted by mast cells fibroblasts, endothelial cells, monocytes and B and T lymphocytes and presents dual anti-inflammatory and pro-inflammatory roles. Many previous works have evidenced its role on capillary leakage in dengue infections [revised by Rachman et al. [35]]. Herein, the IL-10 and IL-6 levels were increased in both DENV-positive groups and the leakage group displayed significant higher levels in comparison to the other groups. Although there was no significant correlation between IL-10 or IL-6 levels and TEER values, as discussed below, the synergistic effect of these cytokines and other immunomediators may explain the stronger inflammatory response and endothelial activation observed in the leakage group.

The CXCL10/IFN-inducible protein 10 (IP-10) is a proinflammatory chemokine secreted by monocytes, endothelial cells and fibroblasts in response to IFN-α,β and IFN-γ. It has chemoattractant activity for cells that express the receptor CXCR3 (mainly activated CD4^+^, memory/activated CD8^+^ and NK cells) and promotes T cell adhesion to endothelial cells [36]. Rathakrishnan and colleagues [25] found higher levels of IP-10 during the febrile phase in patients with secondary dengue in comparison to primary infections. Although they did not observed differences between the groups with and without warning signs at febrile phase, they noticed that IP-10 levels declined steadily for patients without warning signs throughout the disease phases while it remained high in patients with warning signs. In the present work, most of the study participants were experiencing secondary dengue infection, which usually elicits more robust inflammatory responses [37]. We also observed the up-regulation of IP-10 in both DENV-positive groups in comparison to healthy controls. When comparing DENV-positive groups, the group with leakage had significantly higher IP-10 levels. Similar results were obtained previously [38] with elevated serum IP-10 (samples collected within 96 hours of fever onset) in individuals who subsequently developed DHF or Dengue Shock Syndrome (DSS). Ferreira et al [30] and Dejnirattisai et al [39] also found that children with DHF presented higher IP-10 levels than those with DF. Herein, IP-10 serum concentrations appeared to negatively correlate with acquired TEER values indicating its possible role as a vascular permeability inducer. Apanna et al [40] have shown that recombinant IP-10 as well as serum from DENV-positive patients with severe dengue (DHF) induced perturbations of the VE-cadherin-based adherens junction and the tight junction protein ZO-1 of HUVEC monolayers. Interestingly, the association of IP-10 genetic variants with dengue vascular leakage has been shown [41].

IL-1α and IL-1β belong to the IL-1 family that binds to IL-1 receptor (IL-1R) to elicit inflammatory cascade and induce vascular dilation and fever. IL-1RA is a natural antagonist that regulates IL-1α and IL-1β proinflammatory activity [42]. Our results show that IL-1α was significantly elevated in leakage group compared to the no leakage and control groups. Moreover, a negative correlation between IL-1α serum concentrations and TEER readings was noticed. IL-1α had already been shown to increase the in vitro vascular permeability [43].

Granulocyte-monocyte colony stimulating factor (GM-CSF) is produced by a variety of cell types including T cells, macrophages, endothelial cells and fibroblasts upon immune stimulation. In dengue infections, GM-CSF triggers the differentiation of monocytes into inflammatory macrophages, which are one of the main cells producing proinflammatory cytokines, including vascular permeability enhancers, and the major cell targets for DENV replication [44]. Although we did not find any association with disease outcome as previously demonstrated [28] the correlation with TEER might reveal an indirect effect of GM-CSF on plasma leakage through stimulation of stem cells differentiation into monocytes. Increased numbers of monocytes/inflammatory macrophages in turn, would result in higher production of proinflammatory molecules such as TNF-α, IL-6, IL-8, and IL-15. Although only IL-10, IL-6, and IP-10 levels were significantly higher when comparing the leakage and no leakage groups, and only GM-CSF, IL-1α, IL-8, and IP-10 displayed a marked negative correlation with TEER values, the increased levels of these immunomediators may synergistically contribute to endothelial activation. Indeed, core analyses using the IPA predicted a stronger activation of four canonical pathways in the leakage group in comparison to the no leakage group. Those included: 1) Role of Pattern Recognition Receptors in recognition of viruses (z-score = 0.447), which includes the innate immune response via recognition of viral single-stranded RNA by toll-like receptors 7 (TLR-7) and activation of NF-κb transcription factor, resulting in a more robust proinflammatory response (TNF-α, IL-3, IL-5, IL-6, IL-8, IL-12, and IL-13). 2) The triggering receptor expressed on myeloid cells 1 (TREM1) signaling (z-score = 2.333). The activation of this receptor, expressed on neutrophils, monocytes, and macrophages results in upregulation of the inflammatory mediators TNF-α, IL-6, IL-10, IL-8, MCP-3, and MIP-1α. Moreover, IL-1β, IL-6, IL-10, GM-CSF, and TNF-α participate in the TREM-1/TLR cross talk. 3) IL-6 signaling (z-score = 2.646). IL-6 plays a central role in inflammation and cellular immune response. IL-6 binding to Glycoprotein 130 culminates with activation of nuclear factor IL-6, which in turn stimulates the expression of IL-6, IL-8 and VEGF. 4) The acute phase response signaling (z-score = 2.236), based in the upregulation of IL-6, IL-1α, IL-1β, IL-1RA, and TNF-α.

As expected, the main limitation of this study owned the small sample volume available, specially for the individuals with plasma leakage. Because of this, we could not evaluate TEER for all the samples in the groups and also the day of collection could not be the same for all the analyzed samples. Also, dengue-positive patients with normal vital signs and hemogram were discharged from hospital with symptomatic support and medical orientation to return at any worsening sign while dengue-positive patients with impaired vital signs and/or hemogram were admitted to receive intravenous fluids and/or symptomatic support. In the no leakage groups there were patients admitted to the hospital and those who went back home were contacted later to report any worsening symptom. It is unlikely that patients who were discharged and had developed severe dengue have not retuned at the hospital, but we cannot discard the false negative detection of plasma leakage in the no leakage group.

We not included age as a criterion of selection or exclusion of samples. Several studies have demonstrated that children are more susceptible to develop plasma leakage than adults (reviewed in [45]). As expected, we found that mean age of individuals in leakage group (14 years) was lower than that in the no leakage group (36 years) (Table 1).

Although some previous studies had shown influence of viral load on disease severity, high viral loads are not seen in all severe dengue cases (reviewed in [46]). Herein, we found no significant differences in viral load between the DENV infected groups and no correlation between viral load and TEER values. Viremia depends on virus strain, serotype, and immune status of the individual [47]. The time of sampling also influences viral burden as observed by Thomas et al [48] who detected a correlation between increased viral loads and severity of dengue at days 4–6 but not at days 1–3 of disease.

Still, we were able to evaluate groups with similar sample size and find different TEER traces pattern among the three groups in two independent experiments. Principal component and cluster analyses also revealed distinct cytokine profiles among the three tested groups. These results clearly indicated differential interference with endothelial barrier function and levels of immunomediators among the tested groups.

In summary, this work shows that the magnitude of the immune response contributes to the adverse plasma leakage outcomes in patients. Also, serum components are important mediators of changes in endothelial homeostasis during dengue infections characterized by a strong inflammatory response. In particular, the increased levels of IP-10 and the decreased levels of CXCL1 and platelets seem to play a significant role in the disruption of vascular endothelium associated with leakage outcomes after DENV infection.

## Supporting information

S1 Fig TEER changes normalized by albumin levels. The obtained results of percentage of change of normalized resistance at 30 min (A) and 120 min (B) after treatment were divided by albumin levels (g/L) for each serum sample from Healthy blood donors (control, n = 13), DENV infected patients without leakage (no leakage, n = 13), or DENV infected patients with leakage (leakage, n = 13). (*) Asterisks indicate statistically significant differences (ANOVA/ Tukey’s test) between groups with *p*<0.05.
(TIF)

S2 Fig Heat map illustration of expression profile of immunomediators. The concentrations of eight analytes were determined simultaneously using Luminex^®^ 200^™^ System on serum from Healthy blood donors (Ctrl, n = 31), DENV infected patients without leakage (No leakage, n = 29), or DENV infected patients with leakage (Leakage, n = 28). The color scale means the protein expression standard deviations from the mean, with blue for low expression and red for high expression levels.
(TIF)

S3 Fig Correlation between immunomediators levels with TEER. The obtained results of percentage of change of normalized resistance at 30 min (in black) and 120 min (in grey) were correlated with levels of CXCL-1 (A), GM-CSF (B), IL-1α (C), IL-8 (D), and IP-10 (E). *r* = Pearson r correlation coefficient.
(TIF)
