# Supplementary figures and images for: Serum from dengue virus-infected patients with and without plasma leakage differentially affects endothelial cells barrier function in vitro
Source: PLoS One. 2017 Jun 6;12(6):e0178820. doi: 10.1371/journal.pone.0178820 (PMC5460851; doi:10.1371/journal.pone.0178820)

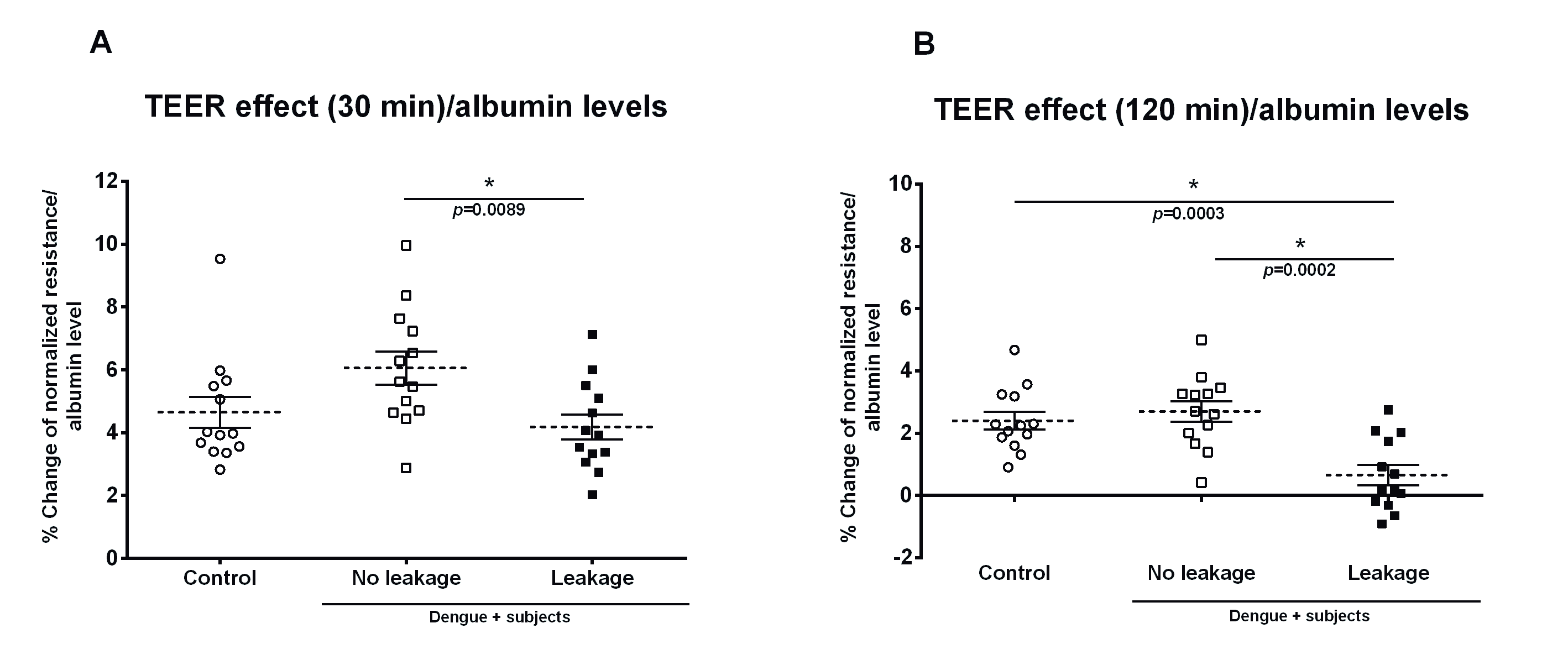

Supplement: S1 Fig — The obtained results of percentage of change of normalized resistance at 30 min (A) and 120 min (B) after treatment were divided by albumin levels (g/L) for each serum sample from Healthy blood donors (control, n = 13), DENV infected patients without leakage (no leakage, n = 13), or DENV infected patients with leakage (leakage, n = 13). (*) Asterisks indicate statistically significant differences (ANOVA/ Tukey’s test) between groups with p<0.05. (TIF) [file pone.0178820.s001.tif]

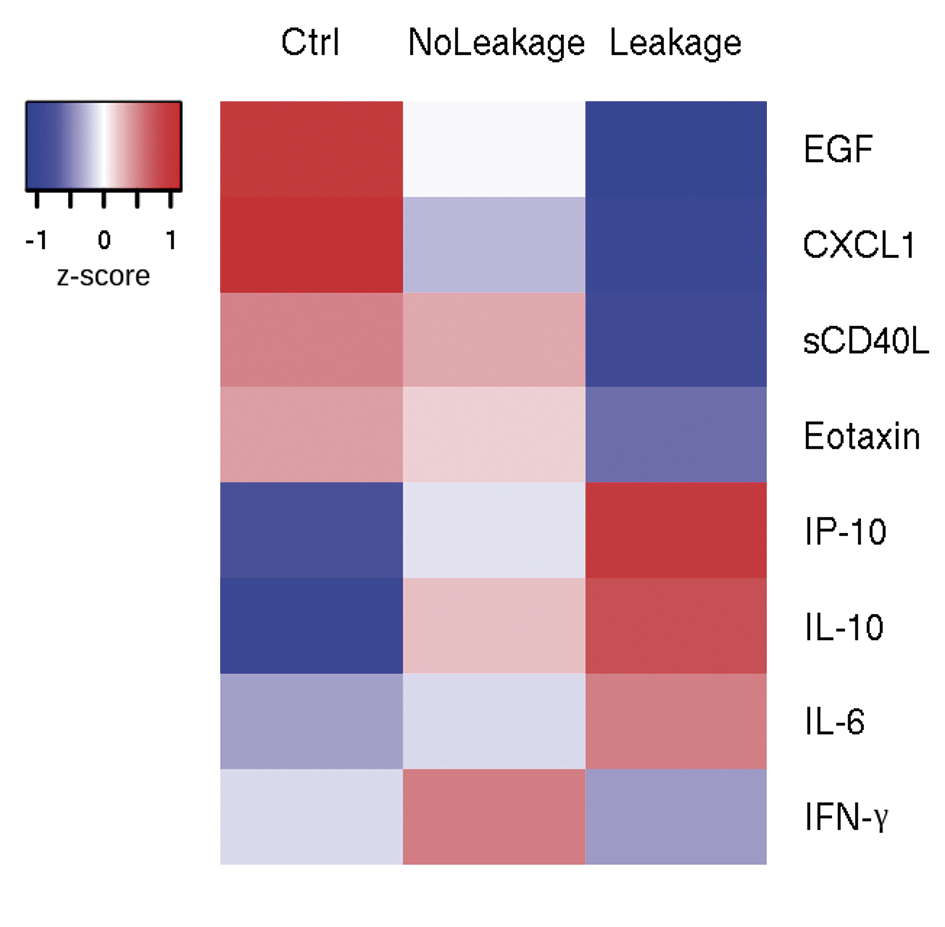

Supplement: S2 Fig — The concentrations of eight analytes were determined simultaneously using Luminex® 200™ System on serum from Healthy blood donors (Ctrl, n = 31), DENV infected patients without leakage (No leakage, n = 29), or DENV infected patients with leakage (Leakage, n = 28). The color scale means the protein expression standard deviations from the mean, with blue for low expression and red for high expression levels. (TIF) [file pone.0178820.s002.tif]

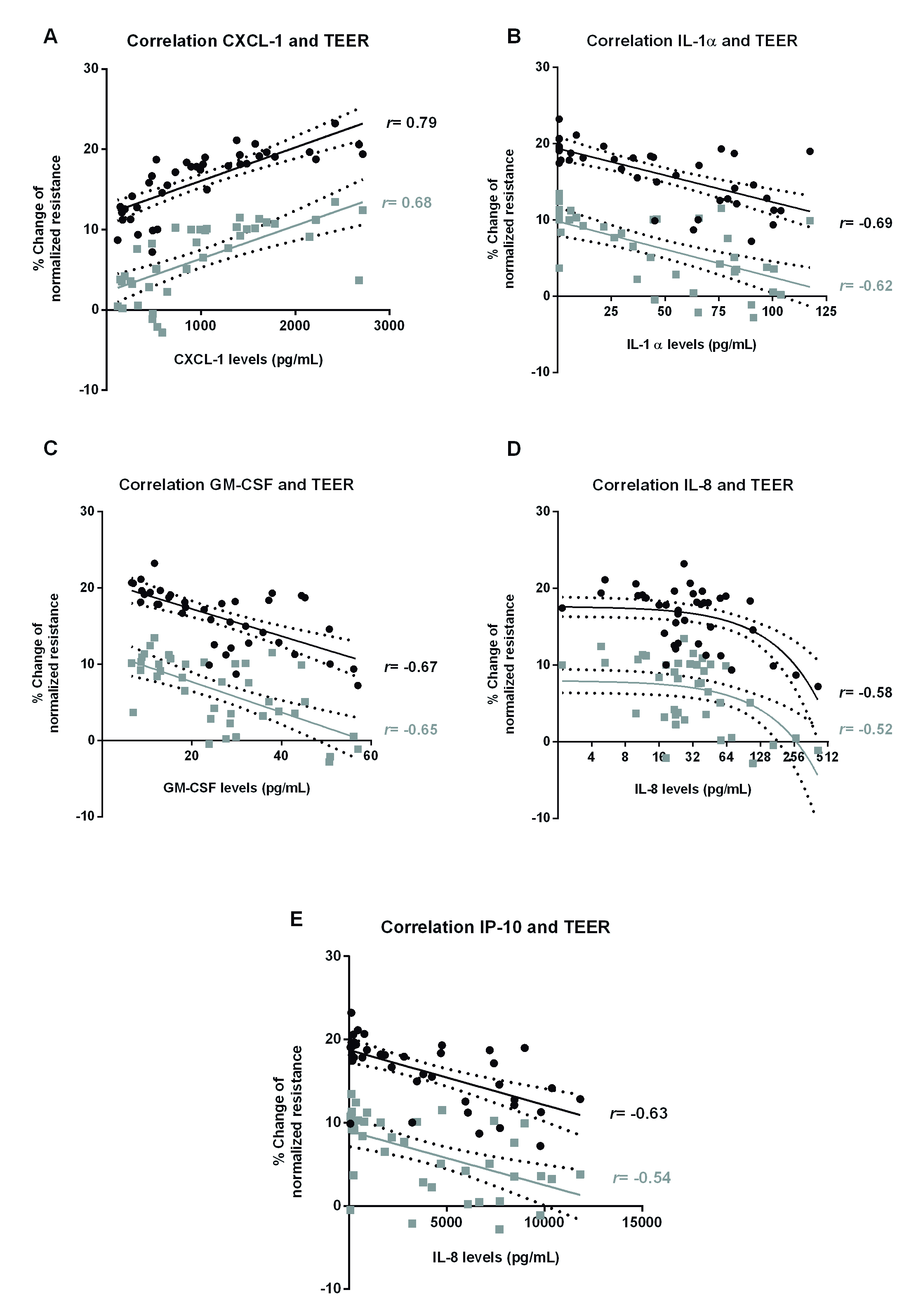

Supplement: S3 Fig — The obtained results of percentage of change of normalized resistance at 30 min (in black) and 120 min (in grey) were correlated with levels of CXCL-1 (A), GM-CSF (B), IL-1α (C), IL-8 (D), and IP-10 (E). r = Pearson r correlation coefficient. (TIF) [file pone.0178820.s003.tif]
